# Does the Polymorphism in the Length of the Polyalanine Tract of* FOXE1* Gene Influence the Risk of Thyroid Dysgenesis Occurrence?

**DOI:** 10.1155/2017/2793205

**Published:** 2017-11-28

**Authors:** Clebson Pantoja Pimentel, Erik Artur Cortinhas-Alves, Edivaldo Herculano Correa de Oliveira, Luiz Carlos Santana-da-Silva

**Affiliations:** ^1^Laboratório de Erros Inatos do Metabolismo, Instituto de Ciências Fisiológicas, Universidade Federal do Pará, Belém, PA, Brazil; ^2^Departamento de Morfologia e Ciências Fisiológicas, Universidade do Estado do Pará, Belém, PA, Brazil; ^3^Laboratório de Cultura de Tecidos e Citogenética, SAMAM, Instituto Evandro Chagas, Ananindeua, PA, Brazil; ^4^Instituto de Ciências Exatas e Naturais, Universidade Federal do Pará, Belém, PA, Brazil

## Abstract

*Background. *Recent data have suggested that polymorphisms in the length of the polyalanine tract (polyA) of* FOXE1* gene may act as a susceptibility factor for thyroid dysgenesis. The main purpose of this study was to investigate the influence of polyA of* FOXE1* gene on the risk of thyroid dysgenesis.* Method. *A case-control study was conducted in a sample of 90 Brazilian patients with thyroid dysgenesis and 131 controls without family history of thyroid disease. Genomic DNA was isolated from peripheral blood samples and the genotype of each individual was determined by automated sequencing.* Results.* More than 90% of genotypes found in the group of patients with thyroid dysgenesis and in controls subjects were represented by sizes 14 and 16 polymorphisms in the following combinations: 14/14, 14/16, and 16/16. Genotypes 14/16 and 16/16 were more frequent in the control group, while genotype 14/14 was more frequent in the group of patients with thyroid dysgenesis. There was no difference between agenesis group and control group. Genotype 14/14 when compared to genotypes 14/16 and 16/16A showed an association with thyroid dysgenesis.* Conclusion.* PolyA of* FOXE1* gene alters the risk of thyroid dysgenesis, which may explain in part the etiology of this disease.

## 1. Introduction

Congenital hypothyroidism (CH) is the most common congenital endocrine disease in childhood and is also a preventable cause of mental deficiency, affecting about one in 3,000–4,000 live births [1, 2]. Thyroid development defects represent 85% of CH cases and are collectively referred to as thyroid dysgenesis (TD), a term that includes agenesis (35–40%), ectopy (30–45%), and hypoplasia (5%) [3, 4]. However, the etiology of TD is still unclear but can be explained on a small scale by genetic mutations in 4 transcription factors (*FOXE1* [5],* NKX2.1* [6],* PAX8* [7, 8], and* NKX2.5* [9]) and at the thyrotrophin receptor [10], which are genes important in the development and normal function of the thyroid gland [11–13].

Among TD-related genes,* FOXE1* gene plays an important role in the migration of precursor cells from thyroid follicles, in addition to being a factor that regulates the transcription of target genes such as thyroglobulin and thyroperoxidase [14].* FOXE1* gene (also called* TTF-2* or* FKHL15*) is located on chromosome 9q22.33 and contains a single exon distributed along 3,461 bp. This gene encodes a 42 kDa protein that contains a forkhead domain and a polyalanine (polyA) tract of variable length (11 to 22 alanine residues) [5, 15, 16]. Polymorphism in the length of the polyA tract in different genes has been associated with different diseases [17, 18]. Studies show that polymorphism in the length of the polyA tract of* FOXE1* gene (rs71369530) is associated with thyroid disorders [19–21].

The mechanism by which variants of the polyA tract act in the CH development have been extensively studied in different populations and results from these studies sometimes differ regarding the influence of the polyA tract on the TD development [18–23], so it is important to know the distribution of mutations and allelic and genotypic frequencies of polymorphisms present in the gene involved in the embryonic development of the thyroid in different populations. Thus, the aim of this work was to investigate variations in the length of the polyA tract of* FOXE1* gene in a group of Brazilian patients with CH in order to better elucidate the potential role of this tract in the genetic susceptibility to TD.

## 2. Subjects and Methods

### 2.1. Sample

Initially, 120 patients with TD were selected through the National Neonatal Screening Program database held at the State University of Pará. All patients came from northern Brazil. To be included in the study, patients should meet the following inclusion criteria: (i) diagnosis of CH according to the biochemical and clinical criteria of the National Neonatal Screening Program (neonatal TSH level less than 10 mUI/L 48 h after birth); (ii) etiological diagnosis of TD type performed through thyroid scintigraphy; (iii) patients could not present a family relationship with each other and (iv) stable use of medication (Levothyroxine). Patients who did not present a conclusive examination of TD type were excluded.

**Figure 1:**
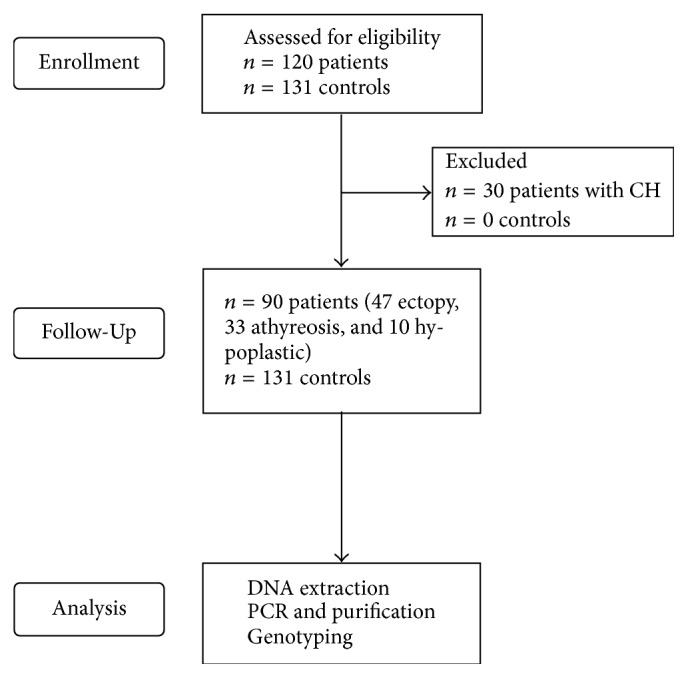
Flowchart diagram of this study.

After observing the inclusion and exclusion criteria, 90 patients (34 males and 56 females) were divided according to TD type. Among patients, 47 (51%) presented ectopy, 33 (38%) agenesis, and 10 (11%) hypoplasia of the thyroid gland. The control group was composed of 131 normal individuals (50 male and 81 female) with no history of CH in the family, who were selected to conduct the case-control association study (Figure 1).

This work is in accordance with the ethical principles that govern human research. The present study was approved by the Ethics Research Committee of the Santa Casa de Misericórdia Foundation Hospital of the State of Pará (2005/0811).

### 2.2. Genotyping

Genomic DNA was isolated from 300 *μ*L of peripheral blood samples using Invisible Spin Blood Mini Kit (Invitrogen of Brazil Ltda., São Paulo, SP, Brazil). The polyA tract of* FOXE1* gene was amplified by polymerase chain reaction (PCR) in final volume of 25 *μ*L, using primes described by Carré et al. (2007) [24]. The PCR mix contained 1x PCR buffer (Invitrogen), 0.2 mM of each dNTP, 1.5 mM MgCl2, 2 pmol of each primer, and 0.1 U of GC rich Taq polymerase (Roche Diagnostics). After an initial denaturation step at 98°C for 30 s, 35 cycles were carried out, each consisting of 1 min at 98°C, primer annealing at 55°C for 30 s, and primer extension of 60 s at 72°C, followed by a final extension of 5 min at 72°C. Amplification was carried out on a gradient thermal cycler (MJ96 +/MJ96G, Biosystems). All PCR products were checked for specificity on a 1.5% agarose gel. Amplicons were purified using PureLink™ PCR Purification kit (Invitrogen of Brazil Ltda., São Paulo, SP, Brazil). The genotypes of the sample studied were determined by direct sequencing using Big Dye Terminator Cycle Sequencing Standart Version 3.1 kit. PCR products were analyzed on an Applied Biosystems sequencer, model ABI 3130 (Applied Biosystems of Brazil Ltda., São Paulo, SP, Brazil).

### 2.3. Statistical Analysis

SPSS software (version 20) was used in data analysis. The *X*^2^ test was used to compare the allelic and genotype frequency of the polyA tract between patients with TD and individuals in the control group and with frequencies found in other studies. The odds ratio (OR) test with a 95% confidence interval was used to evaluate the strength of association between genotypes found and the etiology of TD. The frequencies of genotypes in the length of the polyA tract of* FOXE1* gene are described in the form of absolute and percentage values in Table 1.

## 3. Results

The sequencing analysis of the* FOXE1* gene identified 6 polymorphisms in the length of the polyA tract of* FOXE1* gene that were found in 9 different combinations in patients and controls (Table 1). The gender ratio in both groups did not show significant difference (*X*^2^ = 0.003, *p* = 0.934).

More than 90% of the genotypes found in the TD group (ectopy, agenesis, and hypoplasia) and in the control subjects were represented by sizes 14 and 16 polymorphisms in the following combinations: 14/14, 14/16, and 16/16. Genotype 14/14 was used as a reference for odds ratio calculations for all groups, since this genotype was the most frequent in both TD (ectopy—72.3%, agenesis—66.7% and hypoplasia—70%) and control groups (40.5%). The *X*^2^ test showed a significant difference (*p* < 0.0001) in the proportions of genotypes 14/14, 14/16, and 16/16 when comparing patients with TD and control subjects (Table 2). When compared to genotypes 14/16 and 16/16, genotype 14/14 showed an association with TD (OR = 3.52, 95% CI = 1.95–6.34, *p* < 0.001).

When the proportions of genotypes were analyzed according to the subgroups of ectopy and agenesis, it was possible to observe that genotypes 14/16 + 16/16 were more frequent in the control group (*p* < 0.001 and *p* = 0.005), while genotype 14/14 was more frequent in the TD group (*p* < 0.001). There was no difference between hypoplasia group and control group (*p* = 0.095) (Table 3).

Allele 14 was the most frequent in our sample and in all studies previously published (Hishinuma et al., 2001, Tonacchera et al., 2004; Watkins et al., 2006; Santarpia et al., 2007). However, the allele distribution found in this study was significantly different from the frequencies found in the other studies (Table 4).

## 4. Discussion

The main finding of the present study evidenced that polymorphism in the length of the polyA tract of* FOXE1* gene is associated with genetic susceptibility of TD. Our results indicate that the presence of allele 16 in the polyA tract may be a protective factor against TD compared to genotype 14/14 (OR = 0.28; *p* < 0.001). In addition, it was observed that the presence of allele 16 in the polyA tract decreased the risk for TD, since, in the 16/16 genotype, OR was 0.15 (*p* < 0.001) (Table 2).

Allele 16 has already been described as an important factor to reduce the risk of ectopy [24]. In the sample analyzed in this study, polymorphisms in the length of the polyA tract were associated with the risk of occurrence of TD in the group of patients with ectopy and dysgenesis. Thus, TD is associated with the most common variable (allele 14), as it was possible to evaluate through genotype 14/14, which when compared to genotypes 14/16 and 16/16 shows an association with TD (OR = 3.52; % = 1.95–6.34, *p* < 0.001).

Polymorphism of the polyA tract of other transcription factors has already been associated with more severe clinical findings in other diseases and even to increased morbidity [17, 25]. It is known that the* FOXE1* gene plays an important role in the migration and proliferation of embryonic cells that give rise to the thyroid gland, since the animal model data indicate that mice with knockout of this gene exhibit cleft palate and thyroid malformation (agenesis and ectopy) [7, 26]. Moreover, clinical studies on humans have shown that changes in the structure of* FOXE1* gene (caused by mutations or alterations in the size of the polyA tract) are linked to TD, causing thyroid agenesis [5, 27–29].

The difference in allele frequencies of polymorphisms of the polyA tract can be explained by the different ethnic groups analyzed, especially in our sample, which was composed of population characterized by a high degree of miscegenation between Native Americans, African Americans, and Europeans, a result of the standard of the Brazilian territory colonization [30].

The effects of the polyA tract on the cellular dynamics and the transcription factors are still unclear, because studies have shown conflicting results about the FOXE1 gene with different length of the polyA tract [24, 25, 31–33]. Due to the complexity of thyroid development, it is reasonable to assume that other genes and proteins may have influence on the expression of the FOXE1 gene and its polymorphic variants. However, further studies are necessary to determine it experimentally.

The authors of this work believe that the genetic study of other signaling pathways involved with thyroid formation is important because the morphogenesis of this gland is controlled by a program of gene expression that is ultimately regulated by other transcription factors (*TTF1*,* HHEX*,* NKX2.5,* and* PAX8*), which are organized into a network of genes that, in general, play a role in the proliferation, survival, and migration of thyroid precursor cells [26, 34]. In addition, new studies should be carried out with larger cohorts in order to better understand transmission imbalances, giving greater support to evidence obtained from case-control studies. In conclusion, the most frequent genotypes found were 14/14, 14/16, and 16/16, showing a variation among the studied samples. The variation in the length of the polyA tract of* FOXE1* gene was associated with genetic susceptibility of TD. Allele 16 in the polyA tract may be a protective factor compared to genotype 14/14, decreasing the risk of TD. In addition, when compared to genotypes 14/16 and 16/16 polyA, genotype 14/14 was associated with increased risk of TD.

## Figures and Tables

**Table 1:** Genotypes of DT patients and control subjects according to the polymorphism in the length of the polyA tract of *FOXE1* gene.

Genotypes	Patients				Controls
	Ectopy	Agenesis	Hypoplasia	Total	*n* (%)
	*n* (%)	*n* (%)	*n* (%)	*n* (%)	
11/14	0 (0)	0 (0)	0 (0)	0 (0)	0 (0)
12/14	1 (2.1)	0 (0)	0 (0)	1 (1.1)	2 (1.5)
12/16	0 (0)	1 (3)	0 (0)	1 (1.1)	2 (1.5)
14/14	34 (72.3)	22 (66.7)	7 (70)	63 (70)	53 (40.5)
14/16	10 (21.3)	8 (24.2)	2 (20)	20 (22.2)	49 (37.4)
14/17	0 (0)	1 (3)	0 (0)	1 (1.1)	1 (0.8)
14/19	0 (0)	0 (0)	0 (0)	0 (0)	1 (0.8)
16/16	2 (4.3)	1 (3)	1 (10)	4 (4.4)	22 (16.8)
16/17	0 (0)	0 (0)	0 (0)	0 (0)	1 (0.8)
Total	47 (100)	33 (100)	10 (100)	90 (100)	131 (100)

**Table 2:** Odds ratio of genotypes to the polymorphism in the length of the polyA tract of *FOXE1* gene in all DT patients and control subjects.

Genotypes	Patients	Controls	OR	95% CI	*p* value
	*n* (%)	*n* (%)			
14/14	63 (70)	53 (40.5)	1	—	—
14/16	20 (22.2)	49 (37.4)	0.34	0.18–0.64	<0.001
16/16	4 (4.4)	22 (16.8)	0.15	0.05–0.47	<0.001
14/16 e 16/16	24 (26.7)	71 (54.2)	0.28	0.16–0.51	<0.001
Others	3 (3.3)	7 (5.3)	—	—	—
Total	90 (100)	131 (100)	—	—	—

**Table 3:** Odds ratio of genotypes to the polymorphism in the length of the polyA tract of *FOXE1* gene in DT subgroup (ectopy, agenesis, and hypoplasia) and controls subjects.

Groups (*n*)	Genotypes		Others	OR	95% CI	*p* value
	14/14	14/16 + 16/16				
Ectopy (47)	34 (72%)	12 (26%)	1 (2%)	0.26	0.12–0.56	<0.001
Agenesis (33)	22 (67%)	9 (27%)	2 (6%)	0.31	0.13–0.72	0.005
Hypoplasia (10)	7 (70%)	3 (30%)	0 (0%)	0.32	0.08–1.3	0.095
Controls (131)	53 (41%)	71 (54%)	7 (5%)	—	—	—

**Table 4:** Allele frequency to the polymorphism in the length of the polyA tract of *FOXE1* gene in the present study and literature.

Alleles	Present study		Macchia et al. 1999	Hishinuma et al. (2001)		Polak et al. (2004)		Tonacchera et al. (2004)		Watkins et al. 2006		Santarpia et al. (2007)	
	Brazil		Italy^†^	Japan		France		Italy		New Zealand^†^	Slovenia^†^	Italy	
	TD	C^†^		TD^†^	C	TD^†^	C^†^	TD^†^	C^†^			TD^†^	C
11	1.1	0	0	0.4	0	0	0	0.9	0	0	0	0	0
12	1.1	1.5	2	1.3	0	1	0	0.9	0	0.4	0.6	1.1	0
14	96.7	60.7	54	78.3	97	85.3	65.1	81.6	88.7	67.5	67.2	82.2	94.7
16	1.1	36.6	40	17.4	3	13.7	33.7	16.6	11.3	30	28.4	16.1	5.3
17	0	0.8	4	1.7	0	0	1.2	0	0	0	0	0.6	0
19	0	0.4	0	0.9	0	0	0	0	0	2.1	3.8	0	0

^†^Statistically significant difference in relation to the TD group of the present study [Control (present study)—*p* < 0.0001; Macchia et al.—*p* < 0.0001; TD (Hishinuma et al.)—*p* = 0.0003; TD (Polak et al.)—*p* = 0.0107 and Control (Polak et al.)—*p* = 0.001; TD (Tonacchera et al.)—*p* = 0.0035 and Control (Tonacchera et al.)—*p* = 0.0220; New Zealand (Watkins et al.)—*p* < 0.0001; Slovenia (Watkins et al.)—*p* < 0.0001; DT (Santarpia et al.)—*p* = 0.0018].
